# Supplementary material for: A Potential Health Risk to Occupational User from Exposure to Biocidal Active Chemicals
Source: Int J Environ Res Public Health. 2020 Nov 25;17(23):8770. doi: 10.3390/ijerph17238770 (PMC7728309; doi:10.3390/ijerph17238770)
Supplement: Supplementary file 1 [file ijerph-17-08770-s001.pdf]

Supporting Information(SI) 1. Questionnaire of interview-survey to occupational workers

국립환경과학원
National Institute of Environmental Research

Kstat 케이스탯

○ research institute : National Institute of Environmental Research
○ survey company : K-STAT research

■ in accordance with article 33 of the statistical law, the contents you respond to are strictly confidential and promise not to be used for purpose other than statistical analysis.

Ⅰ. Demographic information

* this is what the interviewer enters.

SQ1. where are you living, city, province?

① seoul ② pusan ③ daegu ④ incheron ⑤ kwangju ⑥ daejun
⑦ wolsan ⑧ kyungi-do ⑨ kwangwon-do ⑩ chungbuk-do ⑪ chungnam-do
⑫ Junbuk-do ⑬ junnam-do ⑭ kyungbuk-do ⑮ kyungnam-do ⑯ Jeju-do

SQ2. what is your age? ________years

SQ2-1. ages datail
① 19year~29year ② 30year ③ 40year ④ 50year +

SQ3. sex ① male ② female

* this is what the interviewer enters.

SQ4. occupational category of respondent, choose occupation.

업종	직업군	업종	직업군
Agriculture occupation/ Forestry occupation	Landscape worker	Medical service related business/Health service business	dental doctor
	채소 및 특용작물 재배원		pharmacy worker
	Crop plantation worker		nurse
	원예작물 재배원		영양사
	조경원		임상병리사
	조림·산림경영인 및 벌목원		x-ray worker
	Forestry-related employee		치과기공사
	제분 및 도정 관련 기계 조작원		치과위생사
	곡물가공 제품 기계 조작원		재활공학 기사
	Agricultural machinery operation employee		물리 및 작업 치료사
	농림·어업 관련 단순 종사원		임상심리사
Fisheries occupation	양식원		Health service business
	Fishery		응급 구조사
	Fishing-related machinery operation employee		위생사
	농림·어업 관련 단순 종사원		의무 기록사
Livestock occupation	Dairy worker		간호조무사
	Livestock breeder	Fabric production and sales business	패턴사
	Livestock industry-related machinery operation employee		재단사
	육류·어패류 및 낙농품 가공 기계 조작원		재봉사
Food cooking and food sales business	Food making & processing related employee		제화원
	Chef & cook		Textile & leather processing worker
	korean food cook		한복 제조원
	chinese food cook		양장 및 양복 제조원
	western food cook		모피 및 가죽 의복 제조원
	japanese food cook		의복·가죽 및 모피 수선원
	beverage cook		기타 의복 제조 관련 기능 종사원
	other food cook		섬유 제조 기계 조작원
	bakery cook & worker		Ironing-related worker
	korean cake cook		shoes making worker
	meat managing worker		Laundry-related worker
	식품 및 담배 등급원	Funeral business, Companion animal business	Veterinarian
	side food cook		장례 상담원 및 장례 지도사
	기타 식품가공 관련 종사원		Pet beauty & care-related worker
	음료 제조 관련 기계 조작원	Children care business	Kindergarten teacher
	Food making machinery operation employee		Child care service worker
	fast food cook		Child daycare teacher
	kitchen related worker		기타 돌봄 및 보건 서비스 종사원
Medical service related business	Medical service related worker		Children's goods rental business worker
	doctor	Beauty/lodging/facility management business	Skin care service worker
	oriental related doctor		Accommodation staff
			Facility management worker/Facility cleaner

from now on, I will ask you about the chemicals product you use in your workplace.

- please respond only to products used at work or workplace, excluding products used at home.
- please respond to your experience of using the product for the past year as of the date of the survey
- the total investigation time does not exceed 30 minutes.

Part A. disinfectant – non-human hygiene disinfectant

A. Have you ever used for last 1year disinfectants in workplaces for past one year?
(<u>살균제</u> 중 귀하께서 **<u>지난 1년간 직장이나 일터에서 사용한 경험이 있는 제품</u>**을 모두 골라주세요.)

NO	product category	use experience	NO	product category	use experience
1	For kitchen	① yes ② no	6	For air-conditioner	① yes ② no
2	For fungi in bathroom	① yes ② no	7	For children's goods	① yes ② no
3	For drainage	① yes ② no	8	For companion animals	① yes ② no
4	For toilet	① yes ② no			
5	For multi-purpose	① yes ② no			

A1-1) 지금부터 지난 1년간 귀하께서 직장이나 일터에서 사용한 경험이 있다고 응답하신 **<u>주방용 살균제</u>**에 대해 여쭙겠습니다.

disinfectant products example for kitchen			
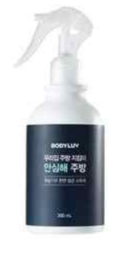	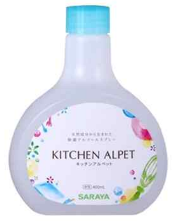	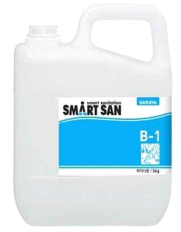	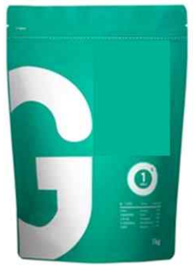

what kinds of disinfectant for kitchen have you used? choose every products (can choose multiple products)

① liquid type	② spray type (trigger)	③ powder type
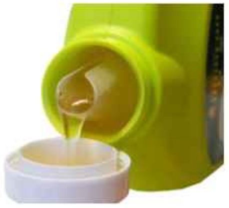	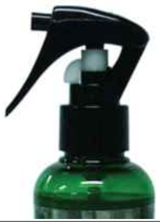	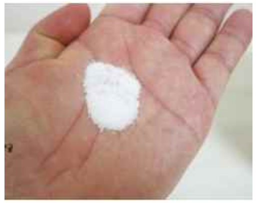

A1-2) **what was products name you used**? size, volume of products, write detail.

application types of products	products name (manufacturing company)<br>[example : - ◇◇ toilet disinfectant (XX company)<br>- ●● disinfecting powder (XX company)]
① liquid type	
② spray type (trigger)	
③ powder type	

A1-3) [use frequency] how often have you used? (A1-1에서 선택한 모든 제품 유형 필수 응답)
*** routine(normal) use in workplace or occupation place (일반적인 사용을 기준으로 응답해 주세요.)**

factors	application types of products	times	use frequency
use frequency	① liquid type	① week ② 1month ③ 6months ④ 1year	( ) times
	② spray type (trigger)	① week ② 1month ③ 6months ④ 1year	( ) times
	③ powder type	① week ② 1month ③ 6months ④ 1year	( ) times

A1-4) [time of use] (문1-1에서 선택한 모든 제품 유형 필수 응답)
*** time of use : mean exposure time from products, chemicals of products when you used products through inhalation and dermal contact**

questions	application types	factors
mean time for using products per one time? (average time taken for disinfecting after spraying and leaving as much disinfectant as desired and then finishing disinfection)	① liquid type	□ min □ sec
mean time for using products per one time? (average time taken for disinfecting after spraying and leaving as much disinfectant as desired and then finishing disinfection)	② spray type (trigger)	□ min □ sec
mean time for using products per one time? (average time taken for disinfecting after using and leaving as much disinfectant as desired and then finishing disinfection)	③ powder type	□ min □ sec

A1-5) [use amount and spaying time] mean used amount and (or) spraying time per use? (문1-1에서 선택한 모든 제품 유형 필수 응답)
*** routine(normal) use in workplace or occupation place (일반적인 사용을 기준으로 응답해 주세요.)**

application types	questions	factors
① liquid type	how many liquor cup(about 50ml) did you used per one use?	① 0.5 cup ② 1cup ③ 1.5cup ④ 2cup ⑤ 2.5cup ⑥ 3cup more( )cup
② spray type (trigger)	number of triggering actions for disinfecting task to kitchen place per one use?	mean □ times
③ powder type	how many spoon(tea spoon) did you used per one use?	mean □ spoon
